# The relationships between social support, medication adherence, and glycemic control among inpatients with type 2 diabetes: a cross-sectional survey in Xi’an, China

**DOI:** 10.3389/fphar.2025.1634768

**Published:** 2025-06-26

**Authors:** Haiyan Li, Hui Min, Lu Zhang, Youjia Li, Jing Wang, Xiaoni Jia

**Affiliations:** ^1^ Department of Pharmacy, Xi’an People’s Hospital (Xi’an Fourth Hospital), Xi’an, China; ^2^ Department of Pharmacy, The First Affiliated Hospital of Xi’an Jiaotong University, Xi’an, China; ^3^ Department of Pharmacy, The Second Affiliated Hospital of Xi’an Jiaotong University, Xi’an, China; ^4^ Department of Endocrinology, Xi’an People’s Hospital (Xi’an Fourth Hospital), Xi’an, China; ^5^ Center Laboratory, Xi’an Mental Health Center, Xi’an, China

**Keywords:** social support, antidiabetic medications, medication adherence, glycemic control, pharmacists, China

## Abstract

**Background:**

Social support may be important in the management of type 2 diabetes. This study aimed to investigate the relationships between social support, medication adherence, and glycemic control in Northwestern China.

**Methods:**

A cross-sectional questionnaire-based survey was carried out in the department of endocrinology in three teaching hospitals between February 2023 and April 2025. The Social Support Rating Scale (SSRS) was used to assess social support. The Adherence to Refills and Medications Scale (ARMS) was used to assess adherence to antidiabetic medications. Logistic regression analyses were performed to identify the factors associated with medication nonadherence and poor glycemic control. Receiver operating characteristic (ROC) was used to assess the performance of the logistic regression model in predicting medication nonadherence.

**Results:**

A total of 522 inpatients finished the questionnaires, 323 (61.9%) inpatients were nonadherent to antidiabetic medications and the prevalence rate of poor glycemic control was estimated to be 82.6%. According to the multivariable logistic regression analysis, inpatients who had received low levels of social support had 2.48 times (95% CI = 1.419–4.322) greater odds of nonadherence to antidiabetic medications than those received high levels of social support, while inpatients who were underweight had 2.78 times (95% CI = 1.054–7.330) greater odds of nonadherence to antidiabetic medications than those with normal BMI. We found that comorbid with hyperlipidemia and combination of oral and injectable antidiabetic medications use were negatively associated with nonadherence to antidiabetic medications. Inpatients prescribed injectable antidiabetic medications were positive associated with poor glycemic control, while older inpatients and the presence of drug-related side effects were negative associated with poor glycemic control.

**Conclusion:**

The relatively low adherence and poor glycemic control among inpatients with T2DM in northwestern China highlighted the urgent need for effective strategies to improve adult diabetes management. Pharmacists should play an important role in strengthening social support to improve adult diabetes self-management.

## Introduction

China has the most people with diabetes, with more than 140 million people estimated in 2021, reaching over 174 million by 2045 (Sun et al., 2022). There is an incredibly heavy economic burden associated with type 2 diabetes mellitus (T2DM) in China, especially in lower income areas (Ding et al., 2022). Drug treatment is currently the main way to treat diabetes, which aimed at maintaining blood glucose levels within a specific range. Optimal glycemic control substantially reduces the risk of diabetes complications and mortality and improves patients’ long-term health and quality of life (Abdissa and Hirpa, 2022; Ghabban et al., 2020). However, glycemic control in individuals with T2DM in developing countries has been persistently poor and is growing steadily worse (Aschner et al., 2020). Treatment success was achieved in only 21% of diabetic patients in China (Strand et al., 2017).

Adherence to medications is defined as the process by which patients take their medications as prescribed. Nonadherence to medications can thus occur in the following situations or combinations thereof: late or non-initiation of the prescribed treatment, suboptimal implementation of the dosing regimen or early discontinuation of the treatment (Vrijens et al., 2012). Higher medication adherence was associated with improved glycemic control (Capoccia et al., 2016; Horii et al., 2019). For each 10% increase in medication adherence, glycosylated hemoglobin (HbA1c) decreases by 0.16% (Schectman et al., 2002). However, the rate of adherence to antidiabetic medications in patients with diabetes is often unsatisfactory (Wong et al., 2015).

Social support referring to the support a patient perceives and receives from his or her social network such as family and friends (Xiao, 1994). In terms of T2DM management, social support can be defined as using social resources for health management behaviors (Glasgow, et al., 2005). Social support is usually measured in the following three dimensions: objective support refers to actual or visible assistance from social networks, subjective support refers to the emotional and subjective experience of being respected, supported, and understood in society, while support utilization includes access and acceptance to various aspects of support and attempts in seeking support from family, relatives, friends, colleagues, and larger community (Xiao, 1994). Social support has been linked to improved medication adherence and glycemic control as well as better T2DM self-management (Dawite et al., 2023; Dedefo et al., 2020; Gu et al., 2017; Huang et al., 2021; Madroumi et al., 2024; Shao et al., 2017). However, previous research focuses on foreign or domestic developed regions. Few studies have fully investigated the relationship between social support, medication adherence, and glycemic control in patients with T2DM in Northwestern China. As the capital city of Shaanxi Province and the largest city in Northwest China, Xi’an is still an area with under- to intermediate-level economic development. With economic development lower than that in the coastal, northeastern and southern regions of China, the area has distinct regional, cultural and lifestyle characteristics, and all of these factors may affect medication adherence and glycemic control among patients with T2DM. Identification of factors associated with medication adherence and glycemic control could help healthcare providers explore strategies to improve health outcomes of patients with diabetes. The objectives of this study were to evaluate the extent of medication adherence and glycemic control among inpatients with T2DM in three large teaching hospitals in Xi’an, China. The factors associated with medication nonadherence and poor glycemic control were investigated, especially the relationships between social support, medication adherence, and glycemic control.

## Materials and methods

### Study design and setting

A questionnaire was constructed and conceptualized based on literature review. Patients admitted to the endocrinology wards of three large tertiary teaching hospitals including Xi’an People’s Hospital (Xi’an Fourth Hospital), the First Affiliated Hospital of Xi’an Jiaotong University and the Second Affiliated Hospital of Xi’an Jiaotong University from February 2023 to April 2025 were eligible to participate in the study. These hospitals are located in Xi’an, Shaanxi Province of Northwestern China and were the highest classification for medical quality given by China’s National Health Commission for all public hospitals.

### Study population and sample size

Inpatients who were more than 18 years old were eligible to participate in the study. The inclusion criteria for participants were inpatients who 1) were diagnosed with T2DM, 2) were currently receiving antidiabetic medications therapy, and 3) with the ability to record medicine information and monitor blood glucose levels, 4) could understand the questionnaire and cooperate with investigators to complete the questionnaire, and 5) agreed to participate in the survey. It should be noted that the study population comprised only patients who might have been on antidiabetic therapy prior to being admitted to the hospital, regardless of when this treatment was initiated. Generally speaking, diabetic patients with elevated blood glucose levels were admitted to the medical wards for treatment. Patients who developed complications of diabetes and experienced drug-related side effects also accounted for a considerable proportion of hospitalized patients. The exclusion criteria were inpatients who 1) were less than 18 years old, 2) were severely ill, 3) had been admitted to the ICU or transferred from or to the ICU halfway, 4) experienced adverse clinical outcomes, including acute cerebral infarction, myocardial infarction or death, during hospitalization, 5) pregnant women, 6) had concurrent malignancy or acute complications, and 7) could not respond due to physical or mental problems.

The minimum number of participants was calculated by using the following formula: *n* = *z* ^2^ *p(1-p)/d* ^2^, where n refers to the sample size, z refers to the coefficient of confidence interval (1.96), p represents the prevalence rate, and d indicates a type I error level of 0.05. According to previous studies, predicted medication adherence for chronic diseases patients was 50% (World Health Organization, 2003). Based on the above assumptions, the minimum sample size was 384 inpatients. A total of 597 respondents agreed to participate in the survey. Thirty-six participants did not return the questionnaire and 39 questionnaires were uncompleted. Ultimately, 522 (87.4%) respondents were recruited in this study and completed the survey.

### Survey procedures

Clinical pharmacists and physicians participated in data collection. All the investigators had received standardized training on survey procedures and communication skills. When the participants encountered difficulties, the investigators were well trained to administer the questionnaires in the same manner of providing assistance, such as detailed explanations and reading items. Participants were approached when they were admitted to the medical wards. The purpose and content of the study were explained to eligible inpatients, and written informed consent was obtained prior to enrollment in this study. A face-to-face interview using a pretested structured questionnaire was conducted, which took approximately 10–15 min to complete. The survey was conducted within 48 h of hospital admission. A pilot study was carried out on 30 participants. Inpatients completed the questionnaire either by themselves or with help from the investigators. For the illiterate subjects, the investigators explained the meaning of the items of the questionnaire and recorded their responses. Participants returned their questionnaires to the investigators immediately after completion in the wards. The questionnaires were checked carefully by the investigators. If there were any errors or missing information, the investigators assisted participants in correcting or filling in the data.

### Measurement instruments

Two validated instruments were used in this study. The Adherence to Refills and Medications Scale (ARMS) was used to assess adherence to antidiabetic medications. The Social Support Rating Scale (SSRS) was used to assess patients’ social support.

### Social Support Rating Scale (SSRS)

Social support was measured with the Chinese version of the Social Support Rating Scale (SSRS) in this study (Xiao, 1994). This scale can be used in the general population for individuals 14 years of age or older. This scale contains 10 items, with three subscales: objective support (3 items), subjective support (4 items), and support utilization (3 items). The scores for objective support, subjective support, and support utilization ranged from 1 to 22, 8 to 32, and 3 to 12, respectively, and the total score ranged from 12 to 66, with higher summary scores indicating stronger social support. Based on previous studies, the total social support score is classified into two levels: low (≤44) and high (>44) (Dai et al., 2016). The SSRS scale is provided as a Supplementary MaterialS1.

### Medication adherence

The ARMS is a validated 12-item scale used to measure adherence to taking and refilling medications among patients with chronic disease (Kripalani et al., 2009). The ARMS comprises two subscales: 8 items about adherence to taking medications and 4 items about refill prescriptions. Each of the 12 items was measured on a four-point Likert-type scale (1 = none of the time, 2 = some of the time, 3 = most of the time, and 4 = all of the time). A lower score, ranging from 12 to 48, represented better adherence. According to the published literature (Kripalani et al., 2009; Polanski et al., 2020), participants were classified into two groups based on their total adherence score: <16 points indicate an adherence group, while ≥16 points indicate a nonadherence group. The Chinese version of the ARMS scale was adapted for use in our study after we obtained authorization from the developers of the scale. The ARMS scale is provided as a Supplementary MaterialS2.

### Data collection

The survey consisted of 5 parts, which included sociodemographic information, the SSRS, the ARMS, clinical data and other information. Sociodemographic characteristics included age, sex, marital status, living status, educational and income level, body mass index (BMI), smoking status, and alcohol consumption status. Age was classified based on literature review. BMI was calculated as weight divided by the square of the height (kg/m^2^). In this study, four categories of the BMI were demonstrated by using the recommended cut-offs in China where BMI less than 18.5 kg/m^2^ indicated underweight, BMI ranged from 18.5 kg/m^2^ to 23.9 kg/m^2^ was for patients with normal BMI, BMI ranged from 24 kg/m^2^ to 27.9 kg/m^2^ indicated overweight whereas BMI ≥28 kg/m^2^ indicated obesity. Clinical data, including comorbidities, medication regimens, medication duration were collected from the hospital information system (HIS) by reviewing the electronic medical records. Laboratory indicators, including HbA1c, fasting plasma glucose (FPG), and postprandial plasma glucose (PPG), were extracted from electronic medical records within 48 h of hospital admission. Antidiabetic therapy regimens were classified as oral antidiabetics, injectable antidiabetic medications (insulin only, combination of insulin and glucagon-like peptide-1 receptor agonists (GLP-1)), and combination of oral antidiabetics and insulin/GLP-1. Other information included drug-related side effects and glucose monitoring status. In addition to the above factors, medication adherence was also included in the evaluation of factors associated with glycemic control. The questionnaire used in this study is provided as a Supplementary MaterialS3.

The questionnaire has demonstrated good reliability and validity. The internal consistency of the questionnaire was evaluated using Cronbach’s α coefficient, which demonstrated acceptable reliability (α = 0.72). We performed confirmatory factor analysis (CFA) using R. The root mean square error of approximation (RMSEA) was 0.089, and the standardized root mean square residual (SRMR) was 0.082, both approaching the acceptable thresholds.

### Glycemic control

HbA1c reflects an individual’s average plasma glucose concentration over the prior 2–3 months. As an indicator of the glycemic control of diabetes patients, HbA1c can be used to evaluate the effectiveness of treatment. Participants’ glycemic control was assessed via the updated HbA1c value recorded in the medical records, where higher value indicated worse condition. Optimal glycemic control was defined as an HbA1c < 7.0% (Xu et al., 2013).

### Outcome measurements

The primary outcome was the extent of medication adherence to antidiabetic medications and glycemic control among inpatients with T2DM in Xi’an. Factors associated with nonadherence to antidiabetic medications and poor glycemic control were investigated as the second outcome in this study, especially the relationships between social support, medication adherence, and glycemic control.

### Statistical analysis

Descriptive statistics were presented as frequencies (percentages) for categorical variables and medians (interquartile ranges) for nonnormally distributed continuous variables. Differences in the candidate variables between nonadherent and adherent inpatients, as well as the candidate variables between inpatients with optimal glycemic control and those with poor glycemic control, were evaluated using the chi-square test for categorical variables, the Mann-Whitney test for skew continuous variables, and the independent sample t-test for normal continuous variables. Univariable and multivariable (all the variables in this study were included) logistic regression analyses were performed to identify the factors associated with medication nonadherent and poor glycemic control. In the adjusted logistic regression model, we adjusted for all covariates using the Enter method. Receiver operating characteristic (ROC) analysis was conducted to assess the performance of the logistic regression model in predicting medication nonadherence. All the statistical analyses were performed using the SPSS V26.0 Statistical Software Package for Windows. A *p* value < 0.05 was considered to indicate statistical significance.

## Results

### General characteristics of participants

The mean age of the 522 participants was 57.9 ± 12.3 years, and the majority (59.8%) were male. A total of 186 (35.6%) inpatients were prescribed oral antidiabetics, while 336 (64.4%) inpatients were prescribed injectable antidiabetic medications (insulin/GLP-1) only or combination of oral and injectable antidiabetic medications. A total of 323 (61.9%) inpatients were nonadherent to antidiabetic medications. The average HbA1c was 9.2% ± 2.4%, and the prevalence rate of poor glycemic control was estimated to be 82.6% (HbA1c ≥ 7.0%) in this study. Majority of the participants (86.2%) reported low levels of social support. The demographic and clinical characteristics of the study subjects are shown in Table 1.

**TABLE 1:** Demographic and clinical characteristics of the study subjects.

Characteristics	Total (n = 522, %)	Adherent (n = 199, %)	Nonadherent (n = 323, %)	P-*value*	Optimal glycemic control (n = 91, %)	Poor glycemic control (n = 431, %)	P-*value*
Age, Median (IQR)	60.0 (51.8, 66.0)	60.0 (53.0, 67.0)	59.0 (50.0, 66.0)	0.095	63.0 (55.5, 68.0)	59.0 (50.0, 66.0)	**0.012**
Age (years)				0.155			0.142
<50	115 (22.0)	35 (17.6)	80 (24.8)		13 (14.3)	102 (23.7)	
50–69	322 (61.7)	129 (64.8)	193 (59.8)		61 (67.0)	261 (60.6)	
≥70	85 (16.3)	35 (17.6)	50 (15.5)		17 (18.7)	68 (15.8)	
Gender				0.456			0.886
Female	210 (40.2)	76 (38.2)	134 (41.5)		36 (39.6)	174 (40.4)	
Male	312 (59.8)	123 (61.8)	189 (58.5)		55 (60.4)	257 (59.6)	
BMI(kg/m^2^)				**0.012**			0.712
<18.5	30 (5.7)	6 (3.0)	24 (7.4)		3 (3.3)	27 (6.3)	
18.5–23.9	239 (45.8)	94 (47.2)	145 (44.9)		44 (48.4)	195 (45.2)	
24–27.9	199 (38.1)	86 (43.2)	113 (35.0)		34 (37.4)	165 (38.3)	
≥28	54 (10.3)	13 (6.5)	41 (12.7)		10 (11.0)	44 (10.2)	
Smoking status				0.203			0.095
Non-smoker	315 (60.3)	127 (63.8)	188 (58.2)		62 (68.1)	253 (58.7)	
Current smoker	207 (39.7)	72 (36.2)	135 (41.8)		29 (31.9)	178 (41.3)	
Alcohol consumption				0.277			0.081
Non-drinker	331 (63.4)	132 (66.3)	199 (61.6)		65 (71.4)	266 (61.7)	
Current drinker	191 (36.6)	67 (33.7)	124 (38.4)		26 (28.6)	165 (38.3)	
Education level				0.264			0.408
≤High school graduation	352 (67.4)	140 (70.4)	212 (65.6)		58 (63.7)	294 (68.2)	
≥University (college) graduation	170 (32.6)	59 (29.6)	111 (34.4)		33 (36.3)	137 (31.8)	
Income/month				0.211			0.156
<4,000 yuan	310 (59.4)	125 (62.8)	185 (57.3)		48 (52.7)	262 (60.8)	
≥4,000 yuan	212 (40.6)	74 (37.2)	138 (42.7)		43 (47.3)	169 (39.2)	
Comorbidities							
Coronary atherosclerotic heart disease	169 (32.4)	63 (31.7)	106 (32.8)	0.783	32 (35.2)	137 (31.8)	0.531
Hyperlipidemia	221 (42.3)	103 (51.8)	118 (36.5)	**0.001**	38 (41.8)	183 (42.5)	0.902
Hypertension	245 (46.9)	89 (44.7)	156 (48.3)	0.427	45 (49.5)	200 (46.4)	0.597
Antidiabetic medications prescribed				0.055			**<0.001**
Oral	186 (35.6)	63 (31.7)	123 (38.1)		57 (62.6)	129 (29.9)	
Insulin ± GLP-1	29 (5.6)	7 (3.5)	22 (6.8)		2 (2.2)	27 (6.3)	
Oral + insulin/GLP-1	307 (58.8)	129 (64.8)	178 (55.1)		32 (35.2)	275 (63.8)	
Medication duration				0.838			0.938
≤5 years	238 (45.6)	94 (47.2)	144 (44.6)		40 (44.0)	198 (45.9)	
6–9 years	71 (13.6)	26 (13.1)	45 (13.9)		13 (14.3)	58 (13.5)	
≥10 years	213 (40.8)	79 (39.7)	134 (41.5)		38 (41.8)	175 (40.6)	
Drug-related side effects				0.092			0.109
No	385 (73.8)	155 (77.9)	230 (71.2)		61 (67.0)	324 (75.2)	
Yes	137 (26.2)	44 (22.1)	93 (28.8)		30 (33.0)	107 (24.8)	
Regular glucose monitoring				0.194			0.090
No	128 (24.5)	55 (27.6)	73 (22.6)		16 (17.6)	112 (26.0)	
Yes	394 (75.5)	144 (72.4)	250 (77.4)		75 (82.4)	319 (74.0)	
Living status				**0.042**			0.416
With family	496 (95.0)	194 (97.5)	302 (93.5)		88 (96.7)	408 (94.7)	
Without family	26 (5.0)	5 (2.5)	21 (6.5)		3 (3.3)	23 (5.3)	
Social Support				**0.001**			0.249
High	72 (13.8)	40 (20.1)	32 (9.9)		16 (17.6)	56 (13.0)	
Low	450 (86.2)	159 (79.9)	291 (90.1)		75 (82.4)	375 (87.0)	

Bold values indicated a p-value <0.05.

Of these 522 inpatients, 91 (17.4%) reached the glycemic target level (HbA1c <7.0%), 139 (26.6%) reached the goal of FPG <7.0 mmol/L, and 157 (30.1%) reached the goal of PPG <11.0 mmol/L. The descriptive statistics of social support, medication adherence and glycemic control are presented in Table 2.

**TABLE 2:** Descriptive statistics of social support, medication adherence and glycemic control.

Variables	x ± s
Social support	34.4 ± 8.3
Objective support	9.3 ± 3.4
Subjective support	18.5 ± 4.9
Support utilization	6.5 ± 2.2
Medication adherence	17.6 ± 4.6
HbA1c (%)	9.2 ± 2.4
FPG	9.2 ± 3.0 mmol/L
PPG	14.4 ± 5.2 mmol/L

HbA1c, glycosylated hemoglobin; FPG, fasting plasma glucose; PPG, postprandial plasma glucose.

### Factors associated with nonadherence to antidiabetic medications

The results of the univariable and multivariable logistic regression analyses of factors associated with nonadherence to antidiabetic medications are provided in Table 3.

**TABLE 3:** Univariable and multivariable logistic regression analyses of factors associated with nonadherence to antidiabetic medications.

Characteristics	Unadjusted OR (95% CI)	P-*value*	Adjusted OR (95% CI)	P-*value*
Age (years)				
<50	1.000(Reference)		1.000(Reference)	
50–69	0.655 (0.415, 1.032)	0.068	0.740 (0.418, 1.311)	0.302
≥70	0.625 (0.348, 1.124)	0.116	0.784 (0.372, 1.652)	0.522
Gender				
Female	1.000(Reference)		1.000(Reference)	
Male	0.871 (0.607, 1.251)	0.456	0.645 (0.383, 1.084)	0.098
BMI(kg/m^2^)				
18.5–23.9	1.000(Reference)		1.000(Reference)	
<18.5	2.593 (1.022, 6.582)	0.045	2.780 (1.054, 7.330)	**0.039**
24–27.9	0.852 (0.581, 1.248)	0.411	0.858 (0.569, 1.296)	0.468
≥28	**2.045 (1.040, 4.018)**	**0.038**	2.086 (0.995, 4.371)	0.052
Smoking status				
Non-smoker	1.000(Reference)		1.000(Reference)	
Current smoker	1.267 (0.880, 1.823)	0.203	1.355 (0.791, 2.319)	0.268
Alcohol consumption				
Non-drinker	1.000(Reference)		1.000(Reference)	
Current drinker	1.228 (0.848, 1.777)	0.277	1.352 (0.806, 2.269)	0.253
Education level				
≤High school graduation	1.000(Reference)		1.000(Reference)	
≥University (college) graduation	1.242 (0.849, 1.819)	0.264	1.219 (0.741, 2.005)	0.435
Income/month				
<4,000 yuan	1.000(Reference)		1.000(Reference)	
≥4,000 yuan	1.260 (0.877, 1.810)	0.211	1.144 (0.707, 1.851)	0.585
Comorbidities				
Coronary atherosclerotic heart disease	1.054 (0.722, 1.539)	0.783	1.125 (0.721, 1.755)	0.604
Hyperlipidemia	0.536 (0.375, 0.768)	**0.001**	0.616 (0.413, 0.918)	**0.017**
Hypertension	1.155 (0.810, 1.646)	0.427	1.241 (0.832, 1.851)	0.290
Antidiabetic medications prescribed				
Oral	1.000(Reference)		1.000(Reference)	
Insulin ± GLP-1	1.610 (0.652, 3.972)	0.302	0.980 (0.371, 2.594)	0.968
Oral + insulin/GLP-1	0.707 (0.484, 1.032)	0.073	0.608 (0.405, 0.913)	**0.016**
Medication duration				
≤5 years	1.000(Reference)		1.000(Reference)	
6–9 years	1.130 (0.653, 1.955)	0.663	1.122 (0.616, 2.044)	0.707
≥10 years	1.107 (0.757, 1.620)	0.600	1.200 (0.772, 1.865)	0.417
Drug-related side effects				
No	1.000(Reference)		1.000(Reference)	
Yes	1.424 (0.943, 2.152)	0.093	1.349 (0.859, 2.120)	0.194
Regular Glucose monitoring				
No	1.000(Reference)		1.000(Reference)	
Yes	1.308 (0.872, 1.962)	0.194	1.107 (0.707, 1.732)	0.657
Living status				
With family	1.000(Reference)		1.000(Reference)	
Without family	2.698 (1.001, 7.274)	0.050	2.354 (0.840, 6.599)	0.103
Social Support				
High	1.000(Reference)		1.000(Reference)	
Low	2.288 (1.383, 3.785)	**0.001**	2.477 (1.419, 4.322)	**0.001**

Bold values indicated a p-value <0.05. OR, odds ratio; CI, confidence interval.

Compared to those received high levels of social support, those received low levels of social support were more likely to be nonadherent to antidiabetic medications (unadjusted odds ratio [OR] and 95% confidence interval [CI] 2.288 [1.383, 3.785]; *p* = 0.001). Inpatients comorbid with hyperlipidemia were less likely to be nonadherent to antidiabetic medications (unadjusted OR 0.536 [0.375, 0.768]; *p* = 0.001). Obese inpatients were more likely to be nonadherent to antidiabetic medications than inpatients with normal BMI (unadjusted OR 2.045 [1.040, 4.018]; *p* = 0.038). According to the multivariable logistic regression analysis, inpatients who had received low levels of social support had 2.48 times (95% CI = 1.419–4.322) greater odds of nonadherence to antidiabetic medications than those received high levels of social support, while inpatients who were underweight had 2.78 times (95% CI = 1.054–7.330) greater odds of nonadherence to antidiabetic medications than those with normal BMI. Comorbid with hyperlipidemia (adjusted odds ratio [OR] and 95% confidence interval [CI], 0.616 [0.413, 0.918]; *p* = 0.017) and combination of oral and injectable antidiabetic medications use (adjusted OR 0.608 [0.405, 0.913]; *p* = 0.016) were negatively associated with nonadherence to antidiabetic medications.

**FIGURE 1:**
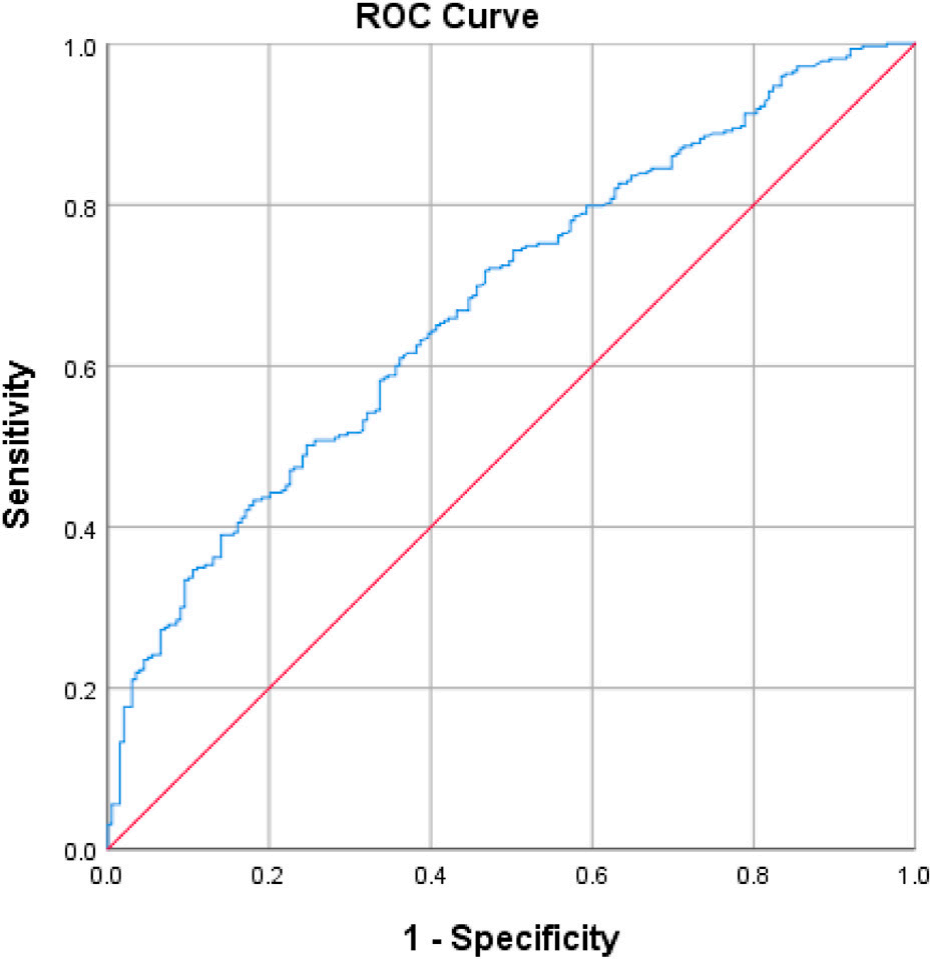
Receiver operating characteristic (ROC) curve for logistic regression model predicting nonadherence to antidiabetic agents: AUC of ROC curve = 0.678 (95% CI = 0.632–0.724). The ROC curve was produced in SPSS.

The receiver operating characteristic (ROC) curve for logistic regression model predicting nonadherence to antidiabetic medications was shown in Figure 1. The model provided an area under the curve (AUC) for the ROC curve of 0.68 (95% CI = 0.63–0.72).

### Factors associated with poor glycemic control

Univariable and multivariable logistic regression analyses of factors associated with poor glycemic control are provided in Table 4.

**TABLE 4:** Univariable and multivariable logistic regression analyses of factors associated with poor glycemic control.

Characteristics	Unadjusted OR (95% CI)	P-value	Adjusted OR (95% CI)	P-value
Age (years)				
<50	1.000(Reference)		1.000(Reference)	
50–69	0.545 (0.287, 1.035)	0.064	0.446 (0.202, 0.984)	**0.046**
≥70	0.510 (0.233, 1.117)	0.092	0.357 (0.132, 0.970)	**0.043**
Gender				
Female	1.000(Reference)		1.000(Reference)	
Male	0.967 (0.609, 1.535)	0.886	0.578 (0.296, 1.127)	0.108
BMI(kg/m^2^)				
18.5–23.9	1.000(Reference)		1.000(Reference)	
<18.5	2.031 (0.590, 6.995)	0.262	1.510 (0.397, 5.752)	0.546
24–27.9	1.095 (0.669, 1.793)	0.718	1.162 (0.667, 2.023)	0.596
≥28	0.993 (0.464, 2.124)	0.985	0.466 (0.193, 1.127)	0.090
Smoking status				
Non-smoker	1.000(Reference)			
Current smoker	1.504 (0.930, 2.433)	0.096	1.502 (0.753, 2.994)	0.248
Alcohol consumption				
Non-drinker	1.000(Reference)		1.000(Reference)	
Current drinker	1.551 (0.946, 2.543)	0.082	1.670 (0.849, 3.285)	0.137
Education level				
≤High school graduation	1.000(Reference)		1.000(Reference)	
≥University (college) graduation	0.819 (0.510, 1.314)	0.408	0.959 (0.515, 1.784)	0.894
Income/month				
<4,000 yuan	1.000(Reference)		1.000(Reference)	
≥4,000 yuan	0.720 (0.457, 1.135)	0.157	0.576 (0.309, 1.071)	0.081
Comorbidities				
Coronary atherosclerotic heart disease	0.859 (0.534, 1.382)	0.532	0.951 (0.540, 1.672)	0.860
Hyperlipidemia	1.029 (0.651, 1.628)	0.902	1.215 (0.711, 2.076)	0.476
Hypertension	0.885 (0.563, 1.391)	0.597	0.971 (0.583, 1.618)	0.911
Antidiabetic medications prescribed				
Oral	1.000(Reference)		1.000(Reference)	
Insulin ± GLP-1	5.965 (1.372, 25.937)	**0.017**	6.695 (1.456, 30.780)	**0.015**
Oral + insulin/GLP-1	3.797 (2.348, 6.142)	**<0.001**	4.491 (2.653, 7.602)	**<0.001**
Medication duration				
≤5 years	1.000(Reference)		1.000(Reference)	
6–9 years	0.901 (0.452, 1.798)	0.768	0.966 (0.450, 2.073)	0.929
≥10 years	0.930 (0.571, 1.516)	0.772	0.878 (0.494, 1.560)	0.657
Drug-related side effects				
No	1.000(Reference)		1.000(Reference)	
Yes	0.672 (0.412, 1.095)	0.110	0.565 (0.323, 0.987)	**0.045**
Regular Glucose monitoring				
No	1.000(Reference)		1.000(Reference)	
Yes	0.608 (0.340, 1.086)	0.093	0.586 (0.307, 1.118)	0.105
Living status				
With family	1.000(Reference)		1.000(Reference)	
Without family	1.654 (0.486, 5.629)	0.421	1.474 (0.398, 5.457)	0.561
Social Support				
High	1.000(Reference)		1.000(Reference)	
Low	1.429 (0.777, 2.625)	0.251	1.218 (0.592, 2.507)	0.591
Medicine adherence				
Adherence	1.000(Reference)		1.000(Reference)	
Non-adherence	1.496 (0.948, 2.361)	0.084	1.681 (0.990, 2.852)	0.054

Bold values indicated a p-value <0.05. OR, odds ratio; CI, confidence interval.

According to the univariable analysis, inpatients who were prescribed injectable antidiabetic medications only (unadjusted OR 5.965 [1.372, 25.937], *p* = 0.017) or combination of oral and injectable antidiabetic medications (unadjusted OR 3.797 [2.348, 6.142], *p* < 0.001) were more likely to report poor glycemic control than those who were prescribed oral antidiabetics. Multivariable logistic regression analyses revealed that inpatients who were prescribed injectable antidiabetic medications only and combination of oral and injectable antidiabetic medications had more than six (adjusted OR 6.695 [1.456, 30.780]; *p* = 0.015) and four (adjusted OR 4.491 [2.653, 7.602]; *p* < 0.001) times greater chance of poor glycemic control, respectively, than inpatients on oral antidiabetic medications alone. Older inpatients (aged 50–69 years; adjusted OR 0.446 [0.202, 0.984]; *p* = 0.046; ≥70 years; adjusted OR 0.357 [0.132, 0.970]; *p* = 0.043) and the presence of drug-related side effects (adjusted OR 0.565 [0.323, 0.987]; *p* = 0.045) were negatively associated with poor glycemic control.

## Discussion

Only 13.8% of the participants received satisfactory social support for diabetes management, which was lower than that previously reported in Thailand (Howteerakul et al., 2007). A total of 199 (38.1%) inpatients were adherent to antidiabetic medications, and 17.4% of inpatients reached the glycemic target level (HbA1c <7.0%) in this study. The prevalence of medication nonadherence in the current study was higher than that documented in the previous studies in China (Huang et al., 2021; Wong et al., 2015; Wu et al., 2023). The prevalence of poor glycemic control among inpatients in the current study was higher than that previously reported in China (Shao et al., 2017; Wang et al., 2021; Wong et al., 2015; Wu et al., 2023; Xiang et al., 2021; Xu et al., 2013), as well as other countries such as Saudi Arabia (Alramadan et al., 2018; Ghabban et al., 2020) and Ethiopia (Dedefo et al., 2020). The mean HbA1c of the participants in our study was 9.2% ± 2.4%, which was higher than 8.4% ± 1.7% reported in Saudi Arabia (Ghabban et al., 2020). The mean FPG was 9.2 ± 3.0 mmol/L in our study, which was lower than 9.9 ± 3.9 mmol/L reported (Ghabban et al., 2020). In this study, 26.6% of the participants had a controlled FPG within 7.0 mmol/L, which indicated superior blood control to that reported in a previous study (19.1%) (Hu et al., 2017). A possible explanation for the poor glycemic control in this study might be that patients with uncontrolled diabetes were more likely to be admitted to the inpatient department for treatment. We only included hospitalized patients in this study, which may have revealed worse condition than that among the general population with diabetes. The results of this study was a warning for healthcare providers in Xi’an to be aware of the suboptimal glycemic control and medication nonadherence in diabetic patients.

Social support was positively associated with medication adherence in this study, which were corresponding to previous studies (Gu et al., 2017; Huang et al., 2021; Khalili Azar et al., 2024; Ramkisson et al., 2017; Shao et al., 2017). Social support is purported to exert its influence in two main ways: (1) directly: providing necessary support to cope with health problems, adhere to self-care regimen and avoid potentially negative situations (for example, economic problems) or (2) indirectly: acting as a protection against the impact of stressful events (Cohen and Wills, 1985). Social support was reported to have a direct effect in a patient’s self-motivation and confidence in managing diabetes, and this improved self-efficacy could translate into improved medical adherence (Shao et al., 2017). It was reported that family and healthcare providers were perceived as important sources of support in promoting medication adherence (Gow et al., 2024). Patients were more likely to be nonadherent when they had less medical knowledge about the medication they had been prescribed (Gow et al., 2024). Patients experiencing poor communication with healthcare providers had higher odds of non-adherence to antidiabetic medications (Gu et al., 2017; Murwanashyaka et al., 2022; Ramkisson et al., 2017). Previous studies emphasized the importance of properly investing efforts in strengthening social support and innovative community care approaches, including pharmacist- and nurse-led care models, which can enhance interventions supporting adult diabetes self-management (Alexandre et al., 2021). Healthcare providers and policy makers need to consider the development of social support programs such as disease management reminders and education with telephone, short message or WeChat (Khalili Azar et al., 2024; Wu et al., 2023; Zhuang et al., 2020) to improve chronic disease management.

Combination of oral and injectable antidiabetic medications use were negatively associated with nonadherence to antidiabetic agents. Our results were in accordance with preceding studies, which indicated that combination of oral antidiabetic medications and insulin use were less likely to be non-adherent than the patients under single oral antidiabetic medications (Murwanashyaka et al., 2022). The literature on the relationship between BMI and medication adherence among patients with diabetes mellitus was particularly scarce. Inpatients who were underweight had 2.78 times (95% CI = 1.054–7.330) greater odds of nonadherence to antidiabetic medications than those with normal BMI in this study. This unexpected result may reflect the complexity of hospitalized patients. Patients with high BMI are generally well aware of the need to strictly follow physical activity and medical nutrition therapy regimens as well as healthy medication adherence behaviors (Hu et al., 2004). However, our findings were inconsistent with previous studies, which indicated that lower BMI was associated with better medication adherence (Marinho et al., 2018). Another study revealed that respondents with normal BMI had higher odds to be non-adherent than those with underweight (Murwanashyaka et al., 2022). Inpatient comorbid with hyperlipidemia were negatively associated with nonadherence to antidiabetic medications. Our results are not in accordance with the previous studies which indicated that patients comorbid with dyslipidemia was more likely to be nonadherent to insulin therapy (He et al., 2017). Another study revealed that patients with better adherence had a better serum lipid profile, particularly higher high-density lipoprotein cholesterol and lower triglycerides levels, than nonadherent patients (Marinho et al., 2018). Further rigorous studies to assess the relationships between BMI and medication adherence, as well as hyperlipidemia and medication adherence in chronic diseases are needed.

Compared to inpatients who were aged younger than 50 years, those who were aged 50 years and older were less likely to report poor glycemic control in this study. The findings of the present study are consistent with those of previously published studies. It has been reported that older patients were less prone to poor glycemic control (Ghabban et al., 2020). A previous study indicated that patients aged 70–79 years were less likely to have poor glycemic control than middle-aged patients (Ki et al., 2014). Another study revealed that patients aged younger than 49 years, 50–59 years and 60–69 years had 3.32 times, 2.61 times and 1.93 times greater odds of having suboptimal glycemic control, respectively, than did those aged 70 years and older (Yeemard et al., 2022). Older diabetic patients are more susceptible to glycemic control, partly because they tend to have more complications with their symptoms, the increased need for higher dosages and intensified medications to control blood glucose (Ki et al., 2014). However, our findings were inconsistent with those of some other studies, which indicated that older patients were significantly more likely to have poor glycemic control. It has been reported that patients aged older than 50 or 65 years have poor glycemic control (Abdissa and Hirpa, 2022; Almetwazi et al., 2019). Inconsistent findings between studies can be partially explained by differences in sample size, study design and methodological issues, as well as age distribution and socioeconomic status of the studied population. The association between age and glycemic control still needs to be further explored in the future.

Inpatients who were prescribed injectable antidiabetic medications (insulin/GLP-1) only or combination of oral and injectable antidiabetic medications were associated with poor glycemic control in this study. These findings were similar to those of previous studies which confirmed that taking insulin (Dedefo et al., 2020; Rossaneis et al., 2019; Wang et al., 2021) or combination of oral antidiabetic medications and insulin use (Ghabban et al., 2020; Nigussie et al., 2021; Wang et al., 2021) was associated with inadequate glycemic control. The use of injectable antidiabetic medications only or combination of oral and injectable antidiabetic medications was reported to be an independent risk factor for inadequate glycemic control among people with T2DM in Saudi Arabia (Alramadan et al., 2018). Insulin use might represent disease severity to some extent. Issues such as difficulty in adhering to regular meals and medication times, fear of hypoglycemia, needles and pain, lack of knowledge of glycemic level and target, and poor self-efficacy with regard to insulin dosage adjustment were found to be barriers to glycemic control in patients using insulin (Tong et al., 2015). Furthermore, the complexity of prescriptions constitutes a burden for patients with diabetes. A previous study revealed that reducing medication regimen complexities might contribute to a significant improvement in HbA1c in patients with diabetes (Abdelaziz and Sadek, 2019). This study demonstrated that inpatients who were prescribed injectable antidiabetic medications should be monitored and followed up to improve glycemic control.

Little is known about the relationship between drug-related side effects and glycemic control among patients with diabetes mellitus in previous studies. The presence of drug-related side effects was negative associated with poor glycemic control in this study. Our finding was not in line with the findings of other studies where drug related problems was positive associated with poor glycemic control (Yimam, Desse and Hebo, 2020). It was reported that drug-related side effects were associated with medication nonadherence (Kassahun et al., 2016; Tominaga et al., 2018). Previous studies indicated that patients with drug related problems often have poor medication adherence that would in turn affect glycemic control (Shareef et al., 2016). When discussing medication instructions, healthcare providers should give due attention to educating patients about the known medication side effects of the prescribed drugs and ways to recognize and cope with them, enabling their confidence in long-term treatment. The relationships between drug-related side effects and glycemic control still needs to be further explored in the future.

Factors affecting glycemic control are multifactorial, as studies have demonstrated, including antidiabetic therapy regimens, adherence to dietary recommendations, physical exercises, and the presence of comorbidities (Dawite et al., 2023; Howteerakul et al., 2007). No significant association was found between social support and glycemic control in this study, which was consistent with those of previous studies (Chew et al., 2015; Ramkisson et al., 2017). It was reported that the impact of social support was only able to explain 8% of the HbA1c variance among T2DM patients in a study in the United States (Latham and Calvillo, 2009). Although family and friends are willing to render support, they may not know how to provide better and more effective support that will positively impact treatment outcomes (Ramkisson et al., 2017). Another possible reason was that family and friends’ support was not a direct pathway for glycemic control (Howteerakul et al., 2007). However, it has also been reported that social support may be a clinically relevant factor on the pathway to glycemic control (Stopford et al., 2013). Family support and composite measures of support were reported most frequently associated with reduced HbA1c (Stopford et al., 2013). It was revealed that the relationship between social support and glycemic control may be mediated by self-efficacy and adherence (Shao et al., 2017). A multitude of social support measures exist with no “gold standard” assessment tool available, which may contribute to these conflicting results (Stopford et al., 2013).

We did not find a significant association between medication adherence and glycemic control in this study. Numerous correlational studies have shown that medication adherence has no significant relationship with glycemic control (Howteerakul et al., 2007; Shrestha et al., 2013; Syafhan et al., 2022). It was reported that only 33% of diabetic patients self-reported to be adherent to therapy were at the treatment goal of FPG ≤7.2 mmol/L (130 mg/dL), and only 20% of patients who were insulin adherent achieved the treatment goal of FPG ≤7.2 mmol/L in a city in northern China (Strand et al., 2017). The findings of this study suggested that the ARMS score should be applied with caution when predicting glycemic control in diabetes patients in clinical practice. However, it has also been reported that higher medication adherence is associated with lower HbA1c levels (Capoccia et al., 2016; Horii et al., 2019; Schectman et al., 2002). Such conflicting opinions might be attributed to differences in socioeconomic status, methodologies employed for adherence assessment, and factors linked to the healthcare system in which the patient receives medical care (Chew et al., 2015).

The findings of this study could help guide the identification of patients who should be monitored and followed up to improve medication adherence and glycemic control. After the patient is admitted to the hospital, the medical staff can ask whether the patient has access to social support sources for diabetes management, such as family support or community care. Patients can be measured with the social support scale if necessary. Appropriate attention should be given to patients who were underweight or with low social support to improve adherence to antidiabetic medications, as well as inpatients who were prescribed injectable antidiabetic medications to improve glycemic control. Pharmacist-led interventions have a significant impact on improving medication adherence and treatment outcomes (Strand et al., 2017; van Eikenhorst et al., 2017; Wu et al., 2023). By collaborating with other healthcare professionals, pharmacists could provide more comprehensive education on diabetes complications, medication, lifestyle, and self-management skills for patient.

### Strengths and limitations

Our study provided valuable insights into diabetes management in similar healthcare settings. Notably, this is one of the few studies examining social support among hospitalized diabetes patients in Northwestern China, addressing a significant research gap in this population. The large sample size, multi-hospital design, use of validated instruments, and comprehensive statistical analysis strengthen the study’s reliability. The findings regarding the importance of social support for medication adherence align well with theoretical expectations and contribute meaningfully to the literature on diabetes care in China. There are several limitations to this study. First, our observation was limited by the fact that it was conducted in Xi’an only. Cultural factors specific to Northwestern China may limit generalizability to other regions. Second, a self-report method was used to assess medication adherence and might be subject to reporting bias. The main disadvantage of self-report questionnaires is the overestimation of adherence. Patients do not usually have sharp recollection to provide correct answers to questionnaires. In addition, they might not provide honest answers for fear of stigmatization (Abdelaziz and Sadek, 2019). Thirdly, the hospitalized patient population likely represents sicker patients, limiting generalizability to outpatient populations. The exclusion of severely ill patients in this study may also limit generalizability. Furthermore, cross-sectional design prevents causal inference and the study period spanning from 2023 to 2025 raises questions about data collection timing. Last but not the least, this study only explored the relationship between medication adherence and glycemic control, but ignored the relationships between diet, physical activity and glycemic control, which was one of the limitations of this study.

## Conclusion and implications

The relatively low adherence and poor glycemic control among inpatients with T2DM in northwestern China highlighted the urgent need for effective strategies to improve disease management. Appropriate attention should be given to inpatients who were underweight or with low social support to improve adherence to antidiabetic medications, as well as inpatients who were prescribed injectable antidiabetic medications to improve glycemic control. Pharmacists should play an important role in improving medication adherence and treatment outcomes.

## Data Availability

The original contributions presented in the study are included in the article/Supplementary Material, further inquiries can be directed to the corresponding authors.
