# Supplementary material for: The relationships between social support, medication adherence, and glycemic control among inpatients with type 2 diabetes: a cross-sectional survey in Xi’an, China
Source: Front Pharmacol. 2025 Jun 26;16:1634768. doi: 10.3389/fphar.2025.1634768 (PMC12240783; doi:10.3389/fphar.2025.1634768)
Supplement: Supplementary file 1 [file Supplementaryfile2.doc]

***ADHERENCE TO REFILLS AND MEDICATIONS SCALE (ARMS)***

*It is common for people to miss taking their medicine from time to time, or to take it differently than prescribed. I would like to ask you about how you actually take your medicines. There are no right or wrong answers. For each question, please answer “none of the time,” “some of the time,” “most of the time,” or “all of the time.”*

**None Some Most All**

1. How often do you forget to take your medicine? 1 2 3 4
2. How often do you decide not to take your medicine? 1 2 3 4
3. How often do you forget to get prescriptions filled? 1 2 3 4
4. How often do you run out of medicine? 1 2 3 4
5. How often do you skip a dose of your medicine before you go to the doctor? 1 2 3 4
6. How often do you miss taking your medicine when you feel better? 1 2 3 4
7. How often do you miss taking your medicine when you feel sick? 1 2 3 4
8. How often do you miss taking your medicine when you are careless? 1 2 3 4
9. How often do you change the dose of your medicines to suit your needs (like 1 2 3 4

when you take more or less pills than you’re supposed to)?

1. How often do you forget to take your medicine when you are supposed to 1 2 3 4

take it more than once a day?

1. How often do you put off refilling your medicines because they cost too much money? 1 2 3 4
2. How often do you plan ahead and refill your medicines before they run out? 4 3 2 1

Copyright © 2009 Emory University

Scoring: Add up the points. The range of possible scores is 12 to 48. Lower scores indicate better adherence.
