# Supplementary material for: The relationships between social support, medication adherence, and glycemic control among inpatients with type 2 diabetes: a cross-sectional survey in Xi’an, China
Source: Front Pharmacol. 2025 Jun 26;16:1634768. doi: 10.3389/fphar.2025.1634768 (PMC12240783; doi:10.3389/fphar.2025.1634768)
Supplement: Supplementary file 2 [file Supplementaryfile1.docx]

**SOCIAL SUPPORT RATING SCALE (SSRS)**

Instructions:

The following questions are asked to describe the support from your family and the society. Please answer them by the best judgment you can make and circle the chosen answer(s). Thanks for your cooperation.

1. How many friends do you consider to be close enough to you that can rely on them for help when you need it?

A. None

B. 1 - 2

C. 3 - 5

D. 6 or more

2. In the last year, you:

A. Stayed away from others and lived alone

B. Moved a lot, and mostly lived with strangers

C. Lived with colleagues, friends, or classmates

D. Lived with your family

3. You and your neighbors

A. Never cared about each other

B. Showed some care when in difficulties

C. Some neighbors cared about you a lot

D. Most of the neighbors cared about you a lot

4. You and your colleagues

1. Never cared about each other

B. Showed some care when in difficulties

C. Some colleagues cared about you a lot

D. Most of the colleagues cared about you a lot

5. Support and care from family members (put a check mark where applicable)

|  | None | Rarely | Some support/care | Strong support/care |
| --- | --- | --- | --- | --- |
| Husband or wife |  |  |  |  |
| Parents |  |  |  |  |
| Children |  |  |  |  |
| Sisters or brothers |  |  |  |  |
| Other family members (e.g., sister-in-law, etc.) |  |  |  |  |

6. In the past, when faced with an emergency, you have received financial or other material support from:

A. None

B. The following (check all that apply):

a. Husband or wife

b. Other family members

c. Friends

d. Relatives

e. Colleagues

f. Employer

g. Union or government

h. Political or religious organizations, society, and nongovernment organization

i. Other (please specify)

7. In the past, when faced with an emergency, you have received console and other emotional support from:

A. None

B. The following (check all that apply):

a. Husband or wife

b. Other family members

c. Friends

d. Relatives

e. Colleagues

f. Employer

g. Union or government

h. Political or religious organization, society, and nongovernment organization

i. Other (please specify)

8. When you feel sad or vexed, you

A. Never talk to anyone

B. Only talk to the closest one or two individuals

C. Will talk to friends if they ask

D. Will talk to friends even if they did not ask

9. When you have difficulties/troubles, you

A. Rely on yourself and do not accept help from others

B. Rarely ask for help

C. Sometime ask for help

D. Always look for help from family members, relatives, and organizations

10. Your participation in activities organized by political or religious organizations, unions, and student associations, etc., can be described as follows:

A. Never

B. Rarely

C. Frequently

D. Always and playing an active roles in these activities
