# Supplementary material for: The relationships between social support, medication adherence, and glycemic control among inpatients with type 2 diabetes: a cross-sectional survey in Xi’an, China
Source: Front Pharmacol. 2025 Jun 26;16:1634768. doi: 10.3389/fphar.2025.1634768 (PMC12240783; doi:10.3389/fphar.2025.1634768)
Supplement: Supplementary file 3 [file Supplementaryfile3.docx]

Hello! These are statements we have made about the antidiabetic agents (oral antidiabetics or injectable antidiabetic agents) prescribed for you. Please truthfully fill in the following information and tick the appropriate option that best suits your situation. There are no right or wrong answers. We are interested in your personal views. Your answer will be kept confidential and will not have any impact on your treatment.

**Age (years):**

**Gender:** A. Female B. male

**Height (m):**

**Weight (kg):**

**Smoking status:**  A. Non-smoker B. Current smoker

**Alcohol consumption:**  A. Non-drinker B. Current drinker

**Education level:**  A. ≤ High school graduation

B. ≥ University (college) graduation

**Income/month:**  A. <4000 yuan B. ≥4000 yuan

**How long have you been taking the antidiabetic agents?**

A. ≤5 years B. 6-9 years C. ≥10 years

**Did you experience drug-related side effects?**  A. No B. Yes

**Regular glucose monitoring:**  A. No B. Yes

**Living status:**  A. With family B. Without family

**ADHERENCE TO REFILLS AND MEDICATIONS SCALE (ARMS)**

It is common for people to miss taking the antidiabetic agents (oral antidiabetics or injectable antidiabetic agents) from time to time, or to take it differently than prescribed. I would like to ask you about how you actually take your antidiabetic agents. There are no right or wrong answers. For each question, please answer “none of the time,” “some of the time,” “most of the time,” or “all of the time.”

1. How often do you forget to take your medicine?

A. None B. Some C. Most D. All

1. How often do you decide not to take your medicine?

A. None B. Some C. Most D. All

1. How often do you forget to get prescriptions filled?

A. None B. Some C. Most D. All

1. How often do you run out of medicine?

A. None B. Some C. Most D. All

1. How often do you skip a dose of your medicine before you go to the doctor?

A. None B. Some C. Most D. All

1. How often do you miss taking your medicine when you feel better?

A. None B. Some C. Most D. All

1. How often do you miss taking your medicine when you feel sick?

A. None B. Some C. Most D. All

1. How often do you miss taking your medicine when you are careless?

A. None B. Some C. Most D. All

1. How often do you change the dose of your medicines to suit your needs

(like when you take more or less pills than you’re supposed to)?

A. None B. Some C. Most D. All

1. How often do you forget to take your medicine when you are supposed to take it

more than once a day?

A. None B. Some C. Most D. All

1. How often do you put off refilling your medicines because they cost too much money?

A. None B. Some C. Most D. All

1. How often do you plan ahead and refill your medicines before they run out?

A. None B. Some C. Most D. All

**Social Support Rating Scale (SSRS)**

The following questions are asked to describe the support from your family and the society. Please answer them by the best judgment you can make and circle the chosen answer(s).

1. How many friends do you consider to be close enough to you that can rely on them for help when you need it?

A. None

B. 1 - 2

C. 3 - 5

D. 6 or more

2. In the last year, you:

A. Stayed away from others and lived alone

B. Moved a lot, and mostly lived with strangers

C. Lived with colleagues, friends, or classmates

D. Lived with your family

3. You and your neighbors

A. Never cared about each other

B. Showed some care when in difficulties

C. Some neighbors cared about you a lot

D. Most of the neighbors cared about you a lot

4. You and your colleagues

1. Never cared about each other

B. Showed some care when in difficulties

C. Some colleagues cared about you a lot

D. Most of the colleagues cared about you a lot

5. Support and care from family members (put a check mark where applicable)

|  | None | Rarely | Some support/care | Strong support/care |
| --- | --- | --- | --- | --- |
| Husband or wife |  |  |  |  |
| Parents |  |  |  |  |
| Children |  |  |  |  |
| Sisters or brothers |  |  |  |  |
| Other family members (e.g., sister-in-law, etc.) |  |  |  |  |

6. In the past, when faced with an emergency, you have received financial or other material support from:

A. None

B. The following (check all that apply):

a. Husband or wife

b. Other family members

c. Friends

d. Relatives

e. Colleagues

f. Employer

g. Union or government

h. Political or religious organizations, society, and nongovernment organization

i. Other (please specify)

7. In the past, when faced with an emergency, you have received console and other emotional support from:

A. None

B. The following (check all that apply):

a. Husband or wife

b. Other family members

c. Friends

d. Relatives

e. Colleagues

f. Employer

g. Union or government

h. Political or religious organization, society, and nongovernment organization

i. Other (please specify)

8. When you feel sad or vexed, you

A. Never talk to anyone

B. Only talk to the closest one or two individuals

C. Will talk to friends if they ask

D. Will talk to friends even if they did not ask

9. When you have difficulties/troubles, you

A. Rely on yourself and do not accept help from others

B. Rarely ask for help

C. Sometime ask for help

D. Always look for help from family members, relatives, and organizations

10. Your participation in activities organized by political or religious organizations, unions, and student associations, etc., can be described as follows:

A. Never

B. Rarely

C. Frequently

D. Always and playing an active roles in these activities
